# An interpretable machine learning model for detecting vision-threatening diabetic retinopathy among patients with diabetic retinopathy: a web-based cross-sectional study

**DOI:** 10.3389/fendo.2026.1776188

**Published:** 2026-03-04

**Authors:** Mingyang Song, Yimeng Shi

**Affiliations:** 1 Department of General Practice, The First Affiliated Hospital of China Medical University, Shenyang, Liaoning, China; 2 Department of Surgery, The First Affiliated Hospital of China Medical University, Shenyang, Liaoning, China

**Keywords:** detection model, machine learning, non-vision-threatening retinopathy, shap, type 2 diabetes, vision-threatening diabetic retinopathy

## Abstract

**Background:**

Vision-threatening diabetic retinopathy (VTDR) is a severe complication of type 2 diabetes mellitus (T2DM), particularly prevalent in patients with prolonged disease duration, poor glycemic control, and systemic comorbidities. This condition frequently progresses asymptomatically toward irreversible blindness without timely intervention. The early identification of VTDR is challenging due to the lack of validated biomarkers and a reliance on subjective clinical assessments. This study aimed to develop and validate an interpretable machine learning (ML) model to detect VTDR among patients with diabetic retinopathy (DR).

**Methods:**

Retrospective clinical data from T2DM patients with DR were extracted from the electronic medical records at our hospital and categorized into VTDR and non-VTDR (defined as mild-to-moderate non-proliferative diabetic retinopathy) groups. The dataset was partitioned into training and testing sets (7:3 ratio). Eight ML models were trained and evaluated using metrics such as Area Under the Curve (AUC), accuracy, and recall. Model performance was evaluated using a comprehensive scoring system (total score = 64). Shapley Additive Explanations (SHAP) were used to interpret the best-performing model. A web-based application was developed to demonstrate potential clinical utility.

**Results:**

Among 1,124 enrolled patients, the prevalence of VTDR was 36.9%. Key associated factors included diabetic treatment, T2DM duration, glycated hemoglobin levels, albuminuria, and anemia. The Support Vector Machine (SVM) model demonstrated superior performance, with an AUC of 0.879, accuracy of 0.837, precision of 0.833, Brier score of 0.129, and an F1 score of 0.756, outperforming the other ML models. The SVM model achieved the highest total score (57/64) in the testing cohort. Furthermore, decision curve analysis and calibration curves confirmed the robustness and reliability of the models. A simplified calculator derived from the SHAP feature importance rankings maintained strong diagnostic capacity.

**Conclusion:**

The interpretable SVM model effectively detected VTDR among patients with DR using routine clinical data. While requiring external validation, this study serves as a proof-of-concept for a cost-effective screening tool that could assist clinicians in prioritizing high-risk patients and facilitating early intervention to prevent irreversible vision impairment.

## Introduction

Diabetic retinopathy (DR), a major microvascular complication of diabetes mellitus, remains the leading cause of preventable vision loss and blindness among the working-age population globally (1). In China alone, epidemiological reports estimate that 19.5 million individuals with diabetes are affected by DR, a significant proportion of whom present with vision-threatening diabetic retinopathy (VTDR) (2). Clinically, VTDR is defined by the presence of severe non-proliferative diabetic retinopathy (severe NPDR), proliferative diabetic retinopathy (PDR), or clinically significant diabetic macular edema (CSME) (3). These pathologies are driven by retinal hypoxia, aberrant angiogenesis, and extracellular fluid accumulation in the macula (4). Consequently, the disease process often evolves subclinically, delaying diagnosis until irreversible structural and functional deficits have occurred. Given the rising global prevalence of type 2 diabetes mellitus (T2DM), VTDR poses a mounting socioeconomic burden, underscoring the urgent need for accessible risk stratification and early intervention strategies (1).

DR progression follows a well-defined trajectory, advancing over years from NPDR to VTDR through sequential pathological changes, including microaneurysm formation, capillary non-perfusion, and progressive retinal ischemia (5). Recent cost-effectiveness analyses suggest that screening intervals should be stratified by risk, proposing quinquennial screening for low-risk patients while advocating for more frequent intervals for high-risk groups (6). While advancements in VTDR screening modalities—such as retinal imaging and optical coherence tomography—have improved diagnostic precision, their widespread implementation remains constrained by systemic limitations, including infrastructural deficits and workforce shortages (7, 8). Furthermore, while deep learning (DL) models based on retinal imagery have achieved high diagnostic performance, they typically require substantial computational power (e.g., high-end GPUs) and large storage capacity. These hardware dependencies create significant barriers to entry in resource-constrained settings, hindering equitable adoption in primary care (9). Compounding these challenges, gaps in understanding VTDR pathogenesis delay targeted therapeutic interventions, leaving many patients with advanced vision deterioration before timely screening (10).

Traditional regression-based prediction models, although inherently interpretable, are constrained by their limited capacity to model the complex non-linear interactions among multifactorial risk variables. This shortfall renders them inadequate for addressing contemporary clinical demands, particularly in heterogeneous patient populations (11). In contrast, artificial intelligence (AI)-driven tools, particularly machine learning (ML), offer transformative potential by deciphering intricate patterns within high-dimensional datasets (12). However, to address the opacity of traditional ML methods, the Explainable AI (XAI) approach, specifically Shapley Additive Explanations (SHAP), provides a cohesive framework for interpreting predictions by quantifying and visually representing the influence of each input feature on model outputs (13). This methodological innovation facilitates the synthesis of demographic, clinical, and biochemical data into personalized diagnostic frameworks, supporting timely and targeted clinical interventions (14). A pivotal advantage of ML models utilizing tabular clinical data lies in their potential for widespread deployment across diverse healthcare settings. Unlike complex image-based systems, these models function effectively even in environments with limited technological infrastructure—a critical prerequisite for expanding equitable screening access to underserved populations (15).

Therefore, this study aimed to (1) develop and validate interpretable ML models for VTDR detection in a high-risk population using real-world data from a Chinese cohort, addressing key challenges such as preprocessing heterogeneous clinical data, optimizing feature selection, and ensuring algorithmic transparency; and (2) rigorously evaluate model performance metrics and elucidate feature contributions using SHAP analysis to align model outputs with clinical reasoning. By integrating interpretable ML with a web-based application, this study advances a proof-of-concept framework for the early identification of VTDR. This approach supports global blindness prevention initiatives by proposing a cost-effective, data-driven, and accessible screening tool designed to facilitate early risk stratification in primary care settings.

## Methods and analysis

### Data source

This retrospective cross-sectional study utilized the electronic medical records (EMRs) of patients diagnosed with DR who visited the Ophthalmology Clinic at The First Affiliated Hospital of China Medical University in Shenyang, China, between May 2021 and February 2025. Consistent with a detection-based framework, this study analyzed data collected at a single time point to identify the presence of VTDR; therefore, no longitudinal follow-up or time-to-event analysis was performed.

The inclusion criteria were: (1) age ≥18 years; (2) a confirmed diagnosis of T2DM; and (3) underwent complete ophthalmic examinations, including fundus photography, with a confirmed diagnosis of DR. The exclusion criteria were: (1) missing data >20%; (2) incomplete or non-gradable fundus imaging; (3) ocular comorbidities unrelated to DR (e.g., cataract, corneal speckles, keratitis); (4) other types of diabetes (e.g., type 1 diabetes mellitus [T1DM], gestational diabetes, secondary diabetes); and (5) presence of acute diabetes complications (e.g., diabetic acidosis, hyperosmolar hyperglycemic state) or concurrent life-threatening conditions (e.g., stroke, myocardial infarction). This study protocol was approved by the Ethics Committee of The First Affiliated Hospital of China Medical University (Approval No. EC-2025-283-2) and was conducted in strict accordance with the principles of the Declaration of Helsinki. The requirement for written informed consent was waived by the institutional ethics committee due to the retrospective nature of the study and the absence of therapeutic intervention. To ensure strict confidentiality, all data were anonymized and de-identified prior to analysis.

### VTDR definition

DR severity was assessed through standardized clinical examinations and bilateral digital fundus photography. Two experienced ophthalmologists, strictly blinded to the patients’ clinical data, independently graded DR severity according to the International Clinical Diabetic Retinopathy (ICDR) severity scale (3). The disease was categorized into five stages: no apparent retinopathy, mild NPDR, moderate NPDR, severe NPDR, and PDR. Diabetic macular oedema (DME) was classified as no DME, non-CSME, or CSME. Based on these assessments, non-VTDR was defined as the presence of mild-to-moderate NPDR, non-CSME, or a combination thereof in either eye. Conversely, VTDR was defined as the presence of severe NPDR, PDR, CSME, or any combination of these conditions in either eye. Patients were excluded if fundus photographs were non-gradable owing to insufficient image quality (e.g., media opacities such as cataracts or vitreous hemorrhage) or missing data. In cases where only one eye met the diagnostic criteria, the final classification was determined using the gradable image from the eligible eye.

### Data collection

Demographic, clinical, and laboratory data were extracted from the EMR system. Demographic and clinical characteristics included age, sex (male/female), body mass index (BMI; kg/m^2^), residence (rural/urban), and educational level (middle school or below/high school or above). Disease-related parameters included T2DM duration (years), smoking status (never/previous/current), alcohol consumption (never/former/current), family history of T2DM, and treatment modality (dietary control, oral medications, insulin injection, or combined oral drugs and insulin). Comorbidities and complications included hypertension, hyperlipidemia, cardiovascular disease, stroke, diabetic nephropathy (DN), diabetic peripheral neuropathy (DPN), lower-extremity arterial disease, anemia, and albuminuria. Ocular parameters included high myopia and a history of eye surgery. Vital signs included systolic blood pressure (SBP) and diastolic blood pressure (DBP; mm Hg). Laboratory indicators included fasting plasma glucose (FPG, mmol/L), glycated hemoglobin (HbA1c; %), blood urea nitrogen (BUN; mmol/L), serum creatinine (SCr; μmol/L), uric acid (UA; μmol/L), total cholesterol (TC; mmol/L), triglycerides (TG; mmol/L), high-density lipoprotein cholesterol (HDL-C; mmol/L), low-density lipoprotein cholesterol (LDL-C; mmol/L), albumin (Alb; g/L), C-reactive protein (CRP; mg/L), aspartate aminotransferase (AST; U/L), alanine aminotransferase (ALT; U/L), white blood cells (WBC; 10^9^/L), platelets (PLT; 10^9^/L), lymphocytes (LYM; 10^9^/L), monocytes (MONO; 10^9^/L), neutrophils (NEUT; 10^9^/L), and neutrophil-to-lymphocyte ratio (NLR). To ensure temporal consistency between candidate variables and retinal outcomes, data extraction was strictly anchored to the date of fundus photography, defined as the “Index Date”. Laboratory indicators and clinical measurements were extracted only if recorded within a strict window of 15 days prior to the Index Date. This window was selected to ensure that physiological data accurately reflected the patient’s status immediately preceding the retinal assessment. To address potential data redundancy, a “nearest-neighbor” prioritization rule was applied: in cases where multiple records existed within this 15-day pre-imaging window, the measurement temporally closest (i.e., most recent) to the Index Date was selected for analysis. Variables with no records in this timeframe were coded as missing and handled via the imputation criteria described below.

### Data preprocessing

To ensure data quality, variables with missing data exceeding 20% were excluded from the analysis (e.g., serum C-peptide, 24h urinary protein). This exclusion threshold was selected based on statistical recommendations to strictly limit the introduction of synthetic noise and bias, particularly for non-routinely measured clinical markers (16). A detailed list of excluded variables and their missing rates is provided in **Supplementary Table 1**. For the remaining variables with minor missingness (<20%), we employed the missForest algorithm for imputation (**Supplementary Figure 1**). Unlike simple mean/mode imputation, missForest is a non-parametric method based on Random Forest. It effectively handles mixed data types (continuous and categorical) inherent in EMRs and captures complex non-linear interactions between variables without making strong distributional assumptions, thereby ensuring reliable data reconstruction for downstream modeling (17).

To prevent distance-based algorithms from being biased by differences in feature magnitudes, all continuous features were standardized using Z-score normalization, scaling them to a mean of 0 and a standard deviation of 1. Categorical variables were encoded based on the specific requirements: One-Hot Encoding for distance-based models (e.g., Support Vector Machine, Neural Network) to avoid introducing artificial ordinal relationships; and Label Encoding for tree-based ensemble models (e.g., Random Forest, Extreme Gradient Boosting) to maintain computational efficiency and preserve the discrete nature of the data.

The dataset was partitioned into training (70%) and testing (30%) sets using random sampling. This stratification ensured that the prevalence of VTDR preserved the class distribution of the original cohort. Addressing the critical concern of data leakage and potential institutional biases, we strictly enforced patient-level separation, ensuring that no individual patient’s data appeared in both the training and testing sets. Furthermore, all preprocessing parameters—including imputation logic (missForest models), Z-score standardization means/standard deviations, and feature scaling boundaries—were fitted solely on the training set. These derived parameters were then applied to transform the testing set. This strict isolation prevents “information leakage”, ensuring that the testing cohort remains a truly unseen dataset and providing a realistic evaluation of model generalization (18).

### Feature selection: the Boruta-LASSO ensemble

To identify robust clinical factors associated with VTDR and reduce dimensionality, feature selection was performed by integrating two complementary methodologies: the Boruta algorithm (19) and Least Absolute Shrinkage and Selection Operator (LASSO) regression (20). The Boruta algorithm, a wrapper method based on Random Forest, was first employed to filter for all relevant features. To establish a baseline for significance, “shadow variables” were constructed by randomly permuting the values of the original features across the samples, thereby destroying their correlation with the outcome while preserving their marginal distribution. The algorithm then trained a Random Forest classifier on the extended dataset (original + shadow features) and compared the importance Z-scores of real features against the maximum Z-score of the shadow features. We performed 100 iterations of this process to ensure stability. Features that consistently showed importance scores higher than the best shadow variable were deemed “confirmed” and retained for the subsequent analysis.

Moreover, to eliminate multicollinearity and refine the feature set, LASSO regression was applied. During 10-fold cross-validation, we selected the regularization parameter lambda (λ).1se—the value within one standard error of the minimum mean cross-validated error—rather than the standard λ.min. This choice was deliberate: given the modest sample size, the λ.1se rule prioritizes a sparser, more parsimonious model, effectively mitigating the risk of overfitting by retaining only the strongest predictors while discarding redundant correlated features (21).

The final feature set was defined as the intersection of variables selected by both algorithms. This strict overlap criterion ensures that selected factors are not only statistically significant (LASSO) but also robust against random noise (Boruta), providing a stable foundation for the subsequent ML modeling (22, 23).

### Model development and validation

Eight ML algorithms—Logistic Regression (LR), Decision Tree (DT), Random Forest (RF), Support Vector Machine (SVM), Neural Network (NNet), Extreme Gradient Boosting (XGB), Light Gradient Boosting Machine (LGB), and CatBoosting (CAT)—were developed to detect VTDR. The specific architectural details used are listed in **Supplementary Table 2**. To ensure optimal performance and prevent overfitting, hyperparameters for each model were tuned using Grid Search with Stratified 5-fold Cross-Validation on the training set. Following rigorous optimization protocols—similar to those established in recent advanced computational frameworks (24, 25)—we explored a comprehensive search space to identify global minima for the loss functions. The exact hyperparameter search spaces and the final optimized hyperparameters for each model are detailed in **Supplementary Table 3**. This transparency ensures reproducibility and confirms that model convergence was achieved.

### Model performance evaluation

Model performance was evaluated using comprehensive metrics: Area Under the Curve (AUC), Accuracy, Sensitivity (Recall), Specificity, Precision, Negative Predictive Value (NPV), F1 score, and Brier score. Formulas for each metric are provided below: Accuracy = (TP + TN)/(TP + TN + FP + FN); Sensitivity = TP/(TP + FN); Specificity = TN/(TN + FP); Precision = TP/(TP + FP); NPV = TN/(TN + FN); F1 score = 2×TP/(2×TP + FP + FN); Brier score (mean squared probability error) = 1/n Σ_i_^n^ (p_i_ – y_i_)² (TP: true positive; TN: true negative; FP: false positive; FN: false negative; n: sample size; y_i_: true label of instance i; p_i_: predicted probability of instance i).

To provide an unbiased, holistic assessment, we employed a rank-based composite scoring system. This method aggregates performance across eight key metrics: AUC, accuracy, sensitivity, specificity, precision, NPV, F1 score, and Brier score (inverted ranking, as lower is better). For each metric, the eight models were ranked from 1 (lowest performance) to 8 (highest performance). These ranks were summed to produce a final Composite Score (Maximum theoretical score = 8 models × 8 metrics = 64). Equal weighting was applied to all metrics to prioritize models with a balanced performance profile rather than those maximizing a single trade-off (e.g., high sensitivity at the cost of low precision) (26).

Calibration curves were generated to evaluate the reliability of risk probabilities. We stratified the predicted probabilities into deciles and plotted the mean predicted probability against the observed fraction of positives. The Brier score was used to quantify the calibration error, where a lower score indicates superior alignment with observed outcomes (27). Additionally, Decision Curve Analysis (DCA) was applied to estimate the net clinical benefit across a wide range of threshold probabilities, determining whether the model offers utility over default strategies (“treat all” or “treat none”) (28). Finally, confusion matrices were generated to visualize the specific trade-offs between false positives and false negatives for each algorithm.

### Model interpretability via SHAP

To provide clinical transparency and interpretability, we employed SHapley Additive exPlanations (SHAP), an XAI framework rooted in cooperative game theory (13). Aligning with emerging standards for “explainable-by-design” medical diagnostics (29), SHAP quantifies the marginal contribution of each individual feature to the model’s outputs. By exhaustively evaluating feature permutations, SHAP assigns values representing both the magnitude (importance) and direction (risk vs. protective) of each feature’s influence on VTDR detection. Crucially, to assess generalization rather than memorization, SHAP values were computed exclusively on the independent testing set. This methodological decision ensures that the explanations reflect the model’s true behavior on unseen data, rather than merely describing the patterns learned during training. This approach provides a clinically accurate interpretation of risk factors, mirroring the rigor required for reliable clinical decision support systems (29).

### Development of a web-based proof-of-concept tool

**Figure 1:**
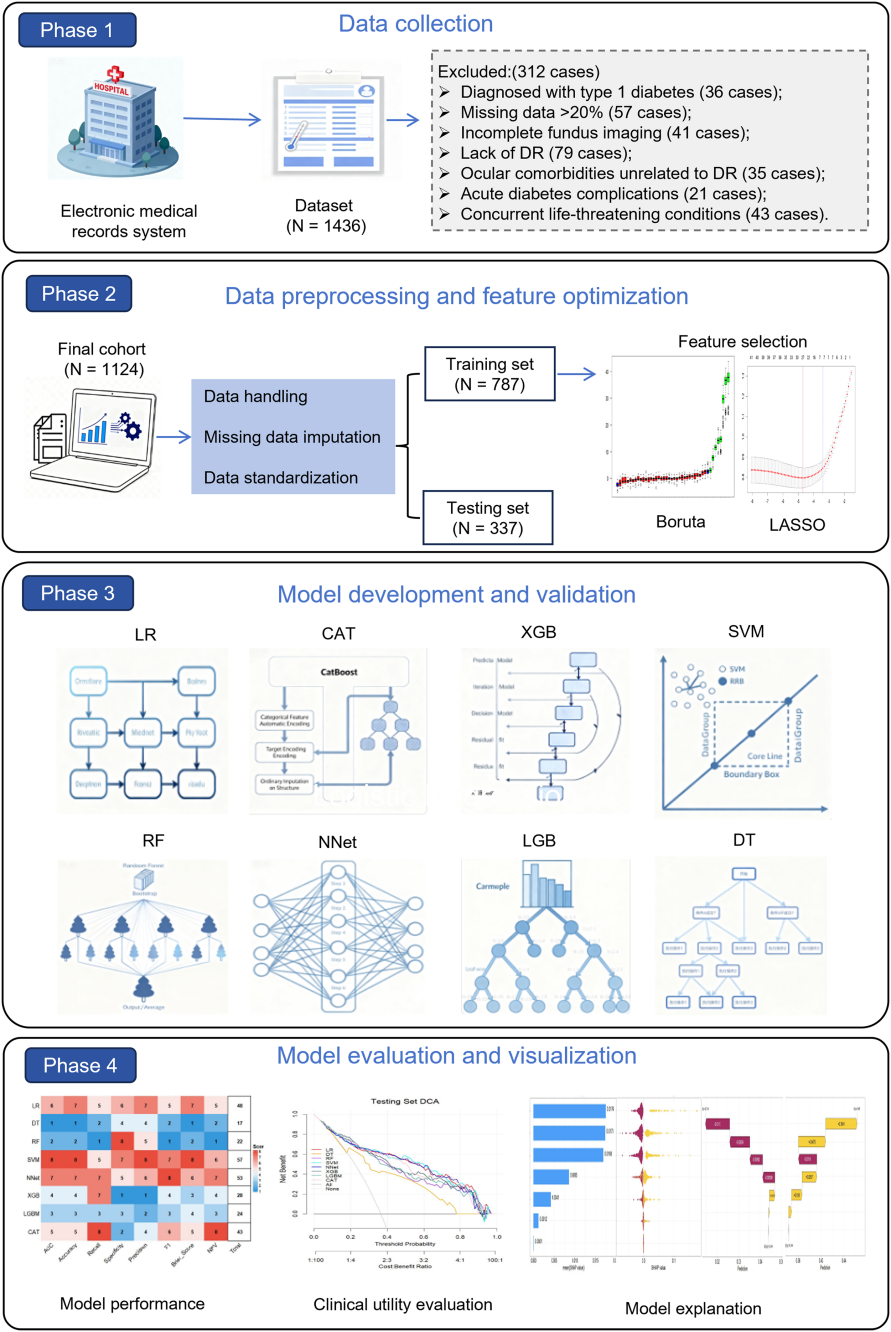
Flowchart of participant selection and study design. LR, Logistic Regression; DT, Decision Tree; RF, Random Forest; SVM, Support Vector Machine; XGB, Extreme Gradient Boosting; LGB, Light Gradient Boosting Machine; NNet, Neural Network; CAT, CatBoosting.

To translate the optimized detection model into a potential clinical utility, we deployed it as an interactive web application using the Shiny framework (RStudio, USA). To ensure data integrity and user safety, the interface implements a dual-layer validation system: categorical variables are captured via standardized drop-down menus to enforce pre-coded options and prevent format errors; continuous variables utilize constrained numeric fields with strict physiological range limits to reject biologically implausible inputs (e.g., negative age or impossible HbA1c levels). The system architecture was optimized for real-time performance on standard hardware, achieving an inference latency of < 1 second. In addition to the risk probability, the output module integrates a “Factor Attribution” table based on SHAP values, providing patient-specific “Feature Contributions” to explain why a specific risk score was assigned. The comprehensive workflow of the study scheme is illustrated in **Figure 1**.

### Statistical analysis

Statistical analyses were performed using IBM SPSS Statistics (version 26.0; IBM Corp.) and the R programming software (version 4.2.3; R Foundation for Statistical Computing, Vienna, Austria). ML models were developed using the “caret” package in R, which provides a standardized framework for training and evaluating diverse algorithms. The following R packages were employed to construct ML models: LR (“glmnet”), DT (“rpart”), RF (“randomForest”), SVM (“e1071”), XGB (“xgboost”), LGB (“lightgbm”), NNet (“nnet”), and CAT (“catboost”).

Continuous variables with non-normally distributed data were reported as medians with interquartile ranges (IQRs) and analyzed using non-parametric tests. The Mann-Whitney U test was used for comparisons. Categorical variables were summarized as frequencies (%) and were evaluated using Pearson’s chi-square test. Statistical significance was determined using a two-tailed *p*-value threshold of < 0.05.

## Results

### Baseline characteristics of the study cohort

Demographic and clinical characteristics of the 1,124 enrolled participants are summarized in **Table 1**. The overall prevalence of VTDR in the cohort was 36.9% (n = 415). Univariate analysis revealed significant differences between VTDR and non-VTDR groups across multiple domains. Specifically, compared with non-VTDR patients, those with VTDR were older (*p* = 0.006), received insulin treatment (*p* < 0.001), had a longer T2DM duration (*p* < 0.001), and exhibited higher rates of comorbidities including diabetic nephropathy (*p* < 0.001), high myopia (*p* = 0.039), anemia (*p* < 0.001), and albuminuria (*p* < 0.001). Furthermore, significant elevations were observed in FPG (*p* = 0.007), HbA1c (*p* < 0.001), SCr (*p* = 0.034), SBP (*p* < 0.001), and DBP (*p* = 0.004) in the VTDR group.

**Table 1:** Baseline characteristics of patients with retinopathy (N = 1,124).

Parameters	Total (N = 1,124)	VTDR (N = 415)	Non-VTDR (N = 709)	Statistics	*p*-value
Age (years)	67.0 [57.0, 75.0]	69.0 [58.0, 76.0]	66.0 [56.0, 74.0]	-2.721	**0.006**
Gender					
Male	686 (61%)	257 (61.9%)	429 (60.5%)	0.222	0.638
Female	438 (39%)	158 (38.1%)	280 (39.5%)		
BMI (kg/m^2^)	23.9 [20.9, 28.4]	24.0 [21.0, 28.4]	23.9 [20.8, 28.4]	-0.131	0.896
Residence					
Rural	481 (42.8%)	171 (41.2%)	310 (43.7%)	0.678	0.410
Urban	643 (57.2%)	244 (58.8%)	399 (56.3%)		
Education level					
middle school or below	640 (56.9%)	234 (56.4%)	406 (57.3%)	0.082	0.774
High school or above	484 (43.1%)	181 (43.6%)	303 (42.7%)		
Duration of diabetes					
<5 years	147 (13.1%)	19 (4.6%)	128 (18.1%)	190.605	**<0.001**
5–10 years	461 (41.0%)	95 (22.9%)	366 (51.6%)		
>10 years	516 (45.9%)	301 (72.5%)	215 (30.3%)		
Smoking status					
Never	737 (65.6%)	269 (64.8%)	468 (66.0%)	0.436	0.804
Former	179 (15.9%)	70 (16.9%)	109 (15.4%)		
Current	208 (18.5%)	76 (18.3%)	132 (18.6%)		
Alcohol consumption					
Never	662 (58.9%)	244 (58.8%)	418 (59.0%)	0.006	0.997
Former	225 (20.0%)	83 (20.0%)	142 (20.0%)		
Current	237 (21.1%)	88 (21.2%)	149 (21.0%)		
Family history of T2DM	547 (48.7%)	201 (48.4%)	346 (48.8%)	0.014	0.905
Eye surgery history	283 (25.2%)	103 (24.8%)	180 (25.4%)	0.045	0.832
Hyperlipidemia	353 (31.4%)	137 (33.0%)	216 (30.5%)	0.788	0.375
Cardiovascular disease	277 (24.6%)	101 (24.3%)	176 (24.8%)	0.033	0.855
Anemia^a^	362 (32.2%)	192 (46.3%)	170 (24.0%)	59.556	**<0.001**
Stroke	129 (11.5%)	49 (11.8%)	80 (11.3%)	0.071	0.790
Diabetic nephropathy	193 (17.2%)	105 (25.3%)	88 (12.4%)	30.578	**<0.001**
Diabetic peripheral neuropathy	148 (13.2%)	56 (13.5%)	92 (13.0%)	0.061	0.804
Lower extremity arterial disease	93 (8.3%)	34 (8.2%)	59 (8.3%)	0.006	0.940
High myopia	147 (13.1%)	43 (10.4%)	104 (14.7%)	4.272	**0.039**
Albuminuria	408 (36.3%)	220 (53.0%)	188 (26.5%)	79.477	**<0.001**
Diabetic treatment					
Dietary control	192 (17.1%)	26 (6.3%)	166 (23.4%)	215.375	**<0.001**
Oral drugs	358 (31.9%)	73 (17.6%)	285 (40.2%)		
Insulin injection	301 (26.8%)	126 (30.4%)	175 (24.7%)		
Oral drugs + insulin	273 (24.3%)	190 (45.8%)	83 (11.7%)		
SBP (mmHg)	136.0 [118.0, 154.0]	145.0 [125.0, 163.0]	131.0 [115.0, 149.0]	-7.397	**<0.001**
DBP (mmHg)	84.0 [75.0, 93.8]	85.0 [76.0, 95.0]	84.0 [74.0, 92.0]	-2.911	**0.004**
Laboratory parameters					
FPG (mmol/L)	7.4 [6.4, 8.5]	7.6 [6.4, 9.8]	7.3 [6.4, 8.2]	-2.681	**0.007**
HbA1c (%)	8.0 [6.2, 9.8]	8.8 [7.3, 11.0]	7.4 [5.5, 9.2]	-10.131	**<0.001**
BUN (mmol/L)	5.7 [4.7, 6.6]	5.8 [4.8, 6.8]	5.7 [4.7, 6.6]	-1.745	0.082
SCr (μmol/L)	78.0 [61.0, 94.0]	81.0 [62.0, 98.0]	77.0 [61.0, 93.0]	-2.129	**0.033**
UA (μmol/L)	325.0 [265.3, 390.8]	330.0 [262.0, 398.0]	323.0 [266.0, 382.0]	-0.689	0.491
TC (mmol/L)	5.2 [4.4, 5.9]	5.2 [4.4, 5.9]	5.2 [4.5, 5.9]	-0.274	0.784
TG (mmol/L)	1.6 [0.7, 2.5]	1.6 [0.7, 2.5]	1.6 [0.8, 2.5]	-0.498	0.619
HDL-C (mmol/L)	1.3 [1.1, 1.5]	1.3 [1.1, 1.5]	1.3 [1.0, 1.5]	-0.643	0.520
LDL-C (mmol/L)	3.0 [2.8, 3.2]	3.0 [2.7, 3.2]	3.0 [2.8, 3.2]	-0.197	0.844
Alb (g/L)	38.0 [33.0, 44.0]	38.0 [33.0, 44.0]	38.0 [33.0, 44.0]	-0.174	0.862
CRP (mg/L)	4.2 [2.3, 5.6]	3.9 [2.2, 5.6]	4.2 [2.3, 5.6]	-0.700	0.484
AST (U/L)	28.0 [22.0, 35.0]	28.0 [22.0, 35.0]	28.0 [21.0, 35.0]	-0.123	0.902
ALT (U/L)	32.0 [26.0, 37.0]	32.0 [26.0, 37.0]	32.0 [26.0, 37.0]	-0.099	0.921
WBC (10^9^/L)	7.0 [5.5, 8.6]	7.0 [5.4, 8.7]	7.1 [5.6, 8.6]	-0.032	0.974
PLT (10^9^/L)	220.0 [159.0, 284.0]	217.0 [160.0, 282.0]	220.0 [157.5, 285.5]	-0.269	0.788
LYM (10^9^/L)	2.4 [1.6, 3.2]	2.4 [1.6, 3.3]	2.3 [1.6, 3.2]	-0.512	0.609
MONO (10^9^/L)	0.6 [0.4, 0.8]	0.6 [0.3, 0.8]	0.6 [0.4, 0.8]	-0.786	0.432
NEUT (10^9^/L)	4.3 [3.1, 5.5]	4.2 [3.1, 5.4]	4.3 [3.1, 5.5]	-1.010	0.312
NLR	1.9 [1.2, 2.9]	1.8 [1.2, 2.8]	1.9 [1.3, 2.9]	-1.072	0.284

^a^ Anemia was defined according to WHO criteria: Hemoglobin <130 g/L for men and <120 g/L for women. To ensure temporal accuracy, this classification was determined dynamically using the specific hemoglobin measurement obtained within the 15-day window immediately preceding the fundus photography. This approach ensures a time-adjusted assessment of the patient’s hematological status concurrent with the retinal examination.

Values are presented as median [Interquartile range] for continuous variables and N (%) for categorical variables. Bold values indicate statistical significance (*p* < 0.05).

BMI, Body Mass Index; T2DM, Type 2 Diabetes Mellitus; SBP, Systolic Blood Pressure; DBP, Diastolic Blood Pressure; FPG, Fasting Plasma Glucose; HbA1c, Glycated Hemoglobin; BUN, Blood Urea Nitrogen; SCr, Serum Creatinine; UA, Uric Acid; TC, Total Cholesterol; TG, Triglycerides; HDL-C, High-Density Lipoprotein Cholesterol; LDL-C, Low-Density Lipoprotein Cholesterol; Alb, Albumin; CRP, C-Reactive Protein; AST, Aspartate Aminotransferase; ALT, Alanine Aminotransferase; WBC, White Blood Cell Count; PLT, Platelet Count; LYM, Lymphocyte Count; MONO, Monocyte Count; NEUT, Neutrophil Count; NLR, Neutrophil-to-Lymphocyte Ratio; VTDR, Vision-Threatening Diabetic Retinopathy.

The study population was randomly divided into a training cohort (n = 787 patients, containing 292 VTDR cases) and a testing cohort (n = 337 patients, containing 123 VTDR cases) in a 7:3 ratio. Statistical analysis revealed no significant differences in baseline characteristics between the training and testing cohorts (*p* > 0.05), ensuring a balanced distribution (**Supplementary Table 4**).

### Optimal feature selection

**Figure 2:**
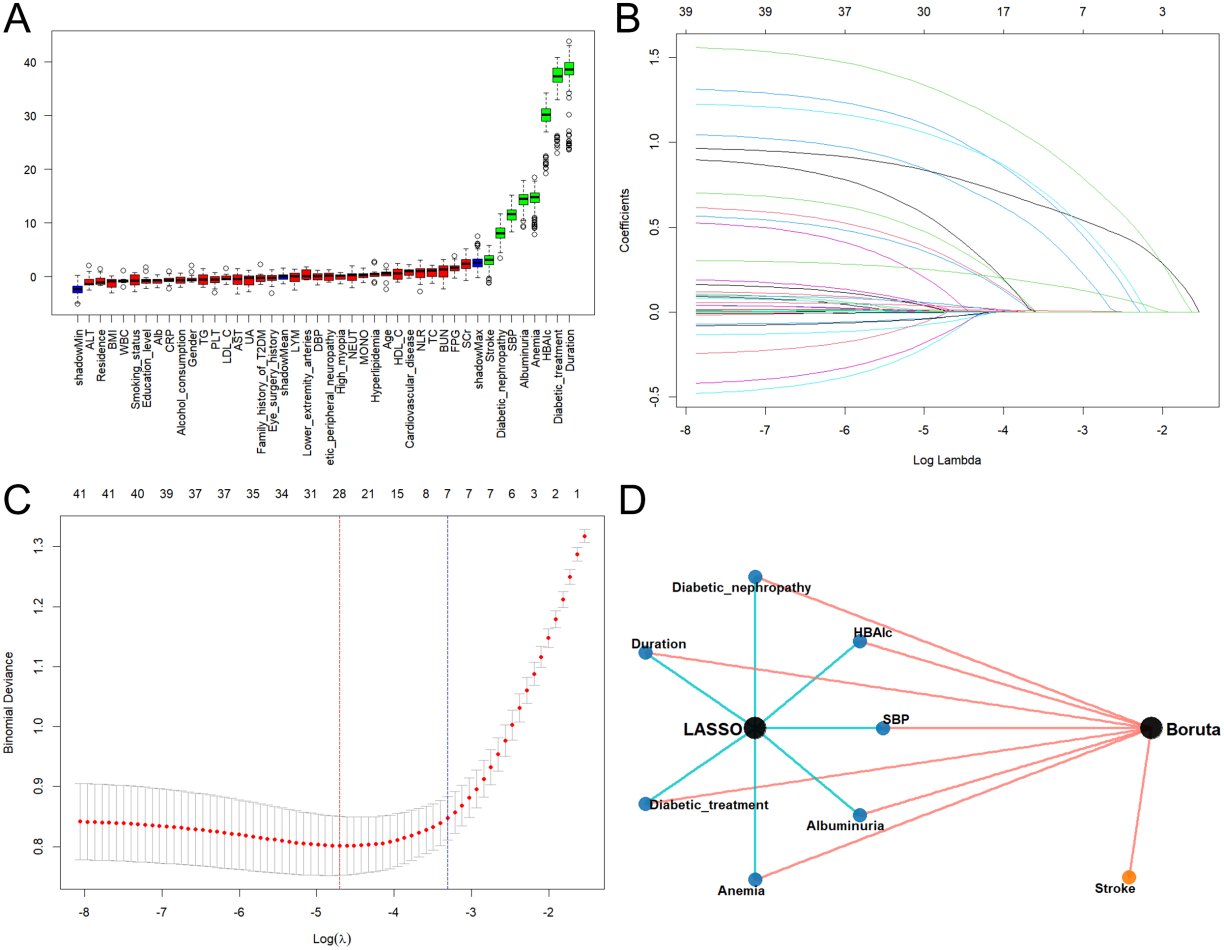
Feature selection using Boruta and LASSO algorithms. **(A)** Boruta algorithm output. Boxplots (blue) depict minimum, mean and maximum Z-scores for shadow attributes. Red and green boxes denote features classified as irrelevant or significant, respectively. **(B)** LASSO coefficient profiles of clinical features. **(C)** Optimal regularization parameter (λ) selection via 10-fold cross-validation. Dashed vertical lines denote the λ values for minimum error (left line) and the one-standard error (1se) rule (right). **(D)** Overlap of features retained by both Boruta and LASSO algorithms. LASSO, Least Absolute Shrinkage and Selection Operator; BMI, Body Mass Index; T2DM, Type 2 Diabetes Mellitus; SBP, Systolic Blood Pressure; DBP, Diastolic Blood Pressure; FPG, Fasting Plasma Glucose; HbA1c, Glycated Hemoglobin; BUN, Blood Urea Nitrogen; SCr, Serum Creatinine; UA, Uric Acid; TC, Total Cholesterol; TG, Triglycerides; HDL-C, High-Density Lipoprotein Cholesterol; LDL-C, Low-Density Lipoprotein Cholesterol; Alb, Albumin; CRP, C-Reactive Protein; AST, Aspartate Aminotransferase; ALT, Alanine Aminotransferase; WBC, White Blood Cell Count; PLT, Platelet Count; LYM, Lymphocyte Count; MONO, Monocyte Count; NEUT, Neutrophil Count; NLR, Neutrophil-to-Lymphocyte Ratio.

Initially, the Boruta algorithm identified eight candidate features with confirmed importance significantly higher than random shadow attributes: T2DM duration, diabetic nephropathy, diabetic treatment, HbA1c, SBP, anemia, stroke, and albuminuria (**Figure 2A**). Subsequently, LASSO regression analysis was employed to refine this feature set. Using 10-fold cross-validation and the rigorous one-standard-error (λ.1se) rule to prioritize model parsimony, LASSO selected seven key variables: T2DM duration, diabetic nephropathy, diabetic treatment, HbA1c, SBP, anemia, and albuminuria (**Figure 2B, C**). Intersection analysis revealed a robust overlap between the two methods (**Figure 2D**). The final feature set consisted of seven consensus variables: T2DM duration, diabetic nephropathy, diabetic treatment, HbA1c, SBP, anemia, and albuminuria. These variables, validated as independent factors associated with the presence of VTDR, were incorporated into the final interpretable ML models.

### Model performance and comparative evaluation

**Figure 3:**
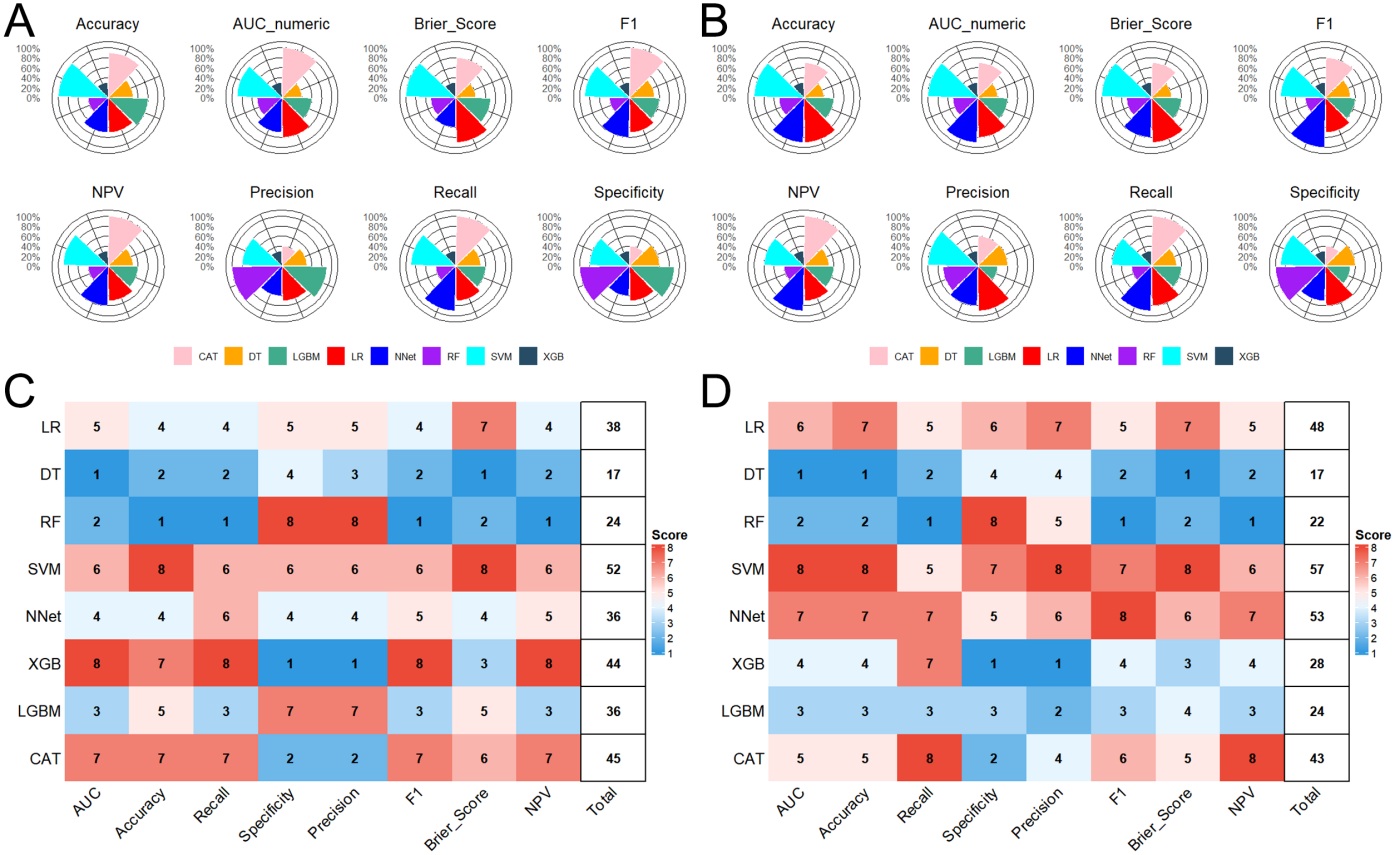
Comparative performance evaluation of eight ML models. **(A, B)** Radar plots illustrating the balance of performance metrics in the training and testing sets. **(C)** and **(D)** Heatmap visualizations of composite metric scores (0–64 scale) across models. Scores reflect holistic model performance across eight metrics, with higher values indicating superior predictive capability. AUC, Area Under the Curve; NPV, Negative Predictive Value; ML, Machine Learning; LR, Logistic Regression; DT, Decision Tree; RF, Random Forest; SVM, Support Vector Machine; XGB, Extreme Gradient Boosting; LGB, Light Gradient Boosting Machine; NNet, Neural Network; CAT, CatBoosting.

**Figure 4:**
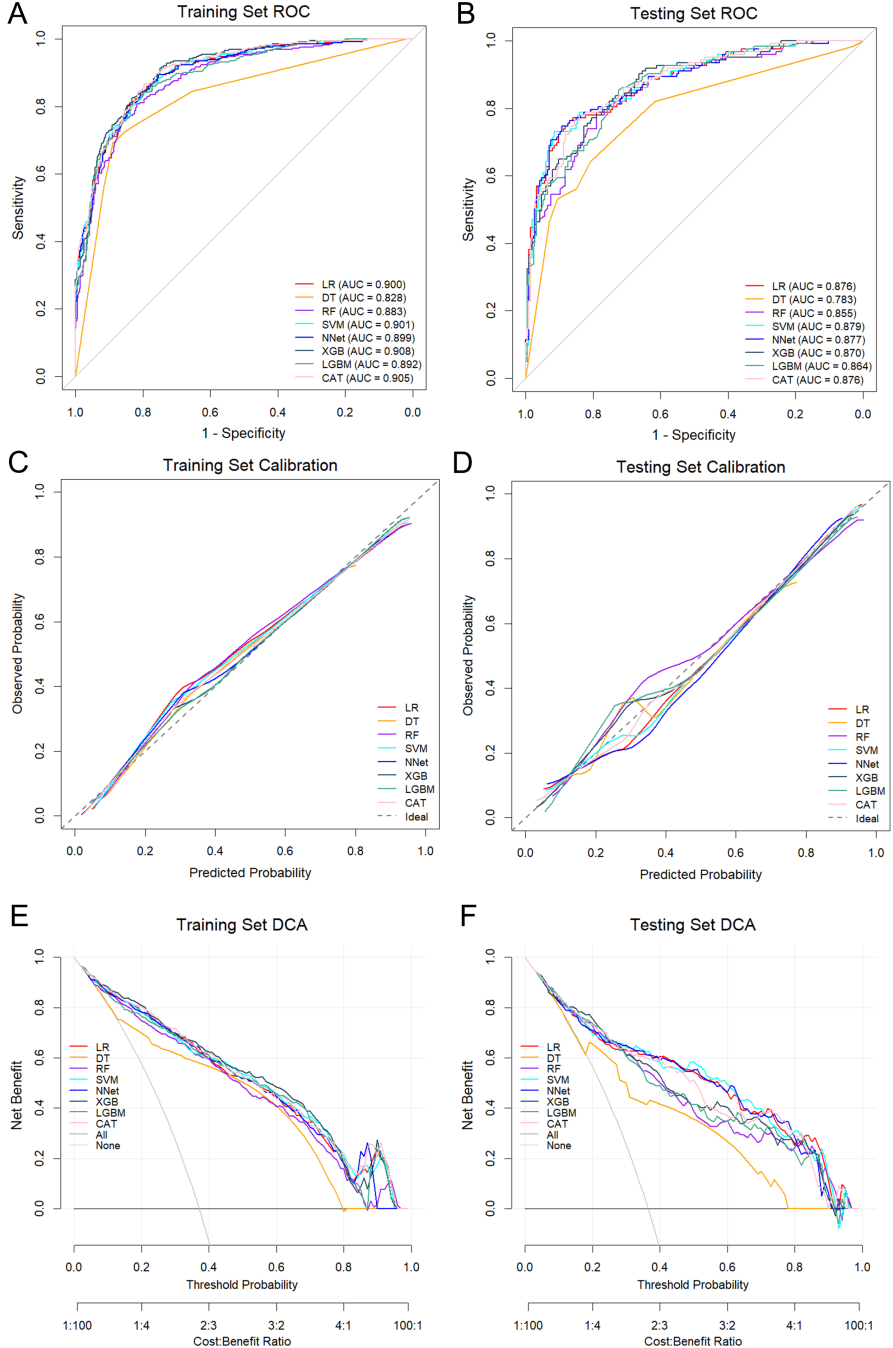
Model discrimination, calibration, and clinical utility. **(A, B)** ROC curves illustrating model discriminative ability. **(C, D)** Calibration curves quantifying the agreement between predicted probabilities and observed outcomes. **(E, F)** DCA evaluating net clinical benefit across a range of threshold probabilities. AUC, Area Under the Curve; ROC, Receiver Operating Characteristic Curve; DCA, Decision Curve Analysis; ML, Machine Learning; LR, Logistic Regression; DT, Decision Tree; RF, Random Forest; SVM, Support Vector Machine; XGB, Extreme Gradient Boosting; LGB, Light Gradient Boosting Machine; NNet, Neural Network; CAT, CatBoosting.

We comprehensively evaluated and compared the performance of eight ML models across both the training and testing cohorts. In the training set, the SVM achieved the highest composite metric score (52/64), indicating superior overall performance (**Figure 3A, C**). Although the XGB model exhibited the strongest discriminative ability with an AUC of 0.908 (95% CI: 0.888–0.929), followed closely by CAT (AUC = 0.905, 95% CI: 0.885–0.926) and SVM (AUC = 0.901, 95% CI: 0.879–0.922) (**Figure 4A**), the SVM maintained the most balanced profile across all evaluated metrics, avoiding the trade-offs seen in other algorithms (**Figure 3C**; **Table 2**). Crucially, in the testing cohort, the SVM model demonstrated robust generalizability, confirming its stability on unseen data. It emerged as the optimal model, attaining the highest composite score (57/64) and outperforming the NNet (53/64) and LR (48/64) models (**Figure 3B, D**). Specifically, the SVM model exhibited the highest discriminative capability (AUC = 0.879; 95% CI: 0.840–0.918) (**Figure 4B**), accompanied by a balanced performance profile, including accuracy (0.837), specificity (0.921), precision (0.833), NPV (0.838), and F1 score (0.756) (**Table 2**).

**Table 2:** Model performance in predicting VTDR in the training and testing sets.

Model	AUC (95% CI)	Recall	Precision	F1 score	Specificity	NPV	Brier score	Accuracy
Training Set								
LR	0.900 (0.878–0.921)	0.712	0.794	0.751	0.891	0.840	0.123	0.825
DT	0.828 (0.798–0.858)	0.692	0.786	0.736	0.889	0.830	0.146	0.816
RF	0.883 (0.859–0.907)	0.620	0.815	0.704	0.917	0.804	0.135	0.807
SVM	0.901 (0.879–0.922)	0.716	0.801	0.756	0.895	0.842	0.122	0.828
NNet	0.899 (0.877–0.921)	0.716	0.792	0.752	0.889	0.841	0.131	0.825
XGB	0.908 (0.888–0.929)	0.812	0.745	0.777	0.836	0.883	0.131	0.827
LGB	0.892 (0.869–0.915)	0.699	0.806	0.749	0.901	0.835	0.128	0.826
CAT	0.905 (0.885–0.926)	0.781	0.760	0.770	0.855	0.869	0.127	0.827
Testing Set								
LR	0.876 (0.837–0.916)	0.691	0.825	0.752	0.916	0.838	0.131	0.834
DT	0.783 (0.732–0.834)	0.537	0.750	0.626	0.897	0.771	0.174	0.766
RF	0.855 (0.813–0.897)	0.512	0.798	0.624	0.925	0.767	0.149	0.774
SVM	0.879 (0.840–0.918)	0.691	0.833	0.756	0.921	0.838	0.129	0.837
NNet	0.877 (0.838–0.917)	0.707	0.813	0.757	0.907	0.844	0.138	0.834
XGB	0.870 (0.831–0.910)	0.707	0.702	0.705	0.827	0.831	0.149	0.783
LGB	0.864 (0.824–0.904)	0.602	0.748	0.667	0.883	0.794	0.143	0.780
CAT	0.876 (0.837–0.914)	0.756	0.750	0.753	0.855	0.859	0.140	0.819

**Figure 5:**
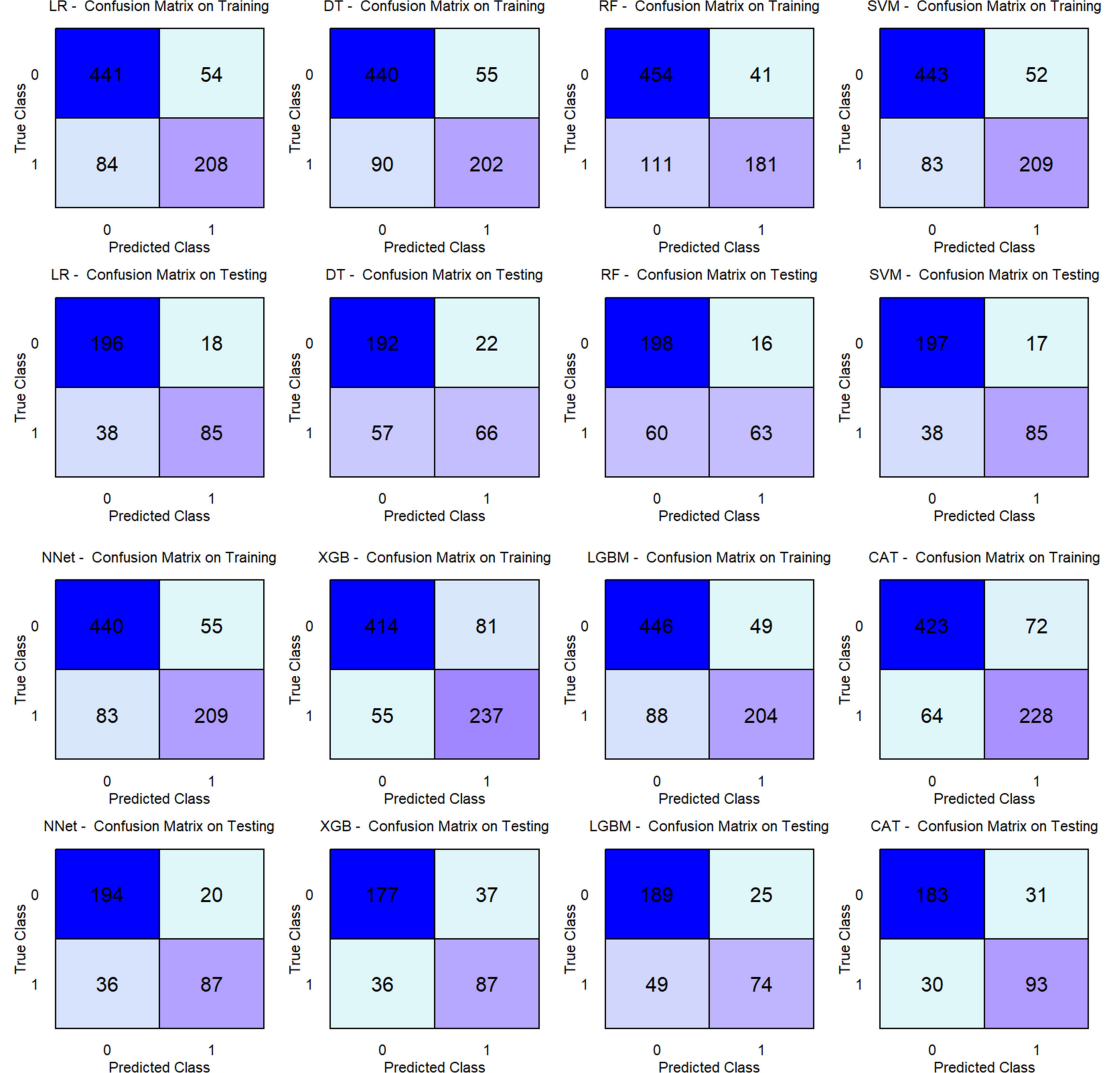
Confusion matrices of eight ML models across two datasets. Each panel displays the counts of True Positives, False Positives, True Negatives, and False Negatives. ML, Machine Learning; LR, Logistic Regression; DT, Decision Tree; RF, Random Forest; SVM, Support Vector Machine; XGB, Extreme Gradient Boosting; LGB, Light Gradient Boosting Machine; NNet, Neural Network; CAT, CatBoosting.

Regarding stratification reliability, calibration curves confirmed that the SVM offered the most accurate probability estimates. It yielded the lowest Brier score in both the training (0.122) and testing (0.129) sets, demonstrating high concordance with the ideal diagonal line (**Figure 4C, D**). Furthermore, DCA was applied to estimate the net clinical benefit. In the training set, the SVM, CAT, and XGB models provided substantial net clinical benefit across a wide threshold probability range (**Figure 4E**). Notably, in the testing cohort, DCA revealed that all models (except DT) offered comparable net clinical utility, with the SVM and RF yielding the highest net benefit across the broadest range of thresholds (**Figure 4F**). Confusion matrix analysis further corroborated the robustness of the SVM model (**Figure 5**). The model demonstrated a balanced distribution of true positives (n = 85) and true negatives (n = 197), ensuring that the majority of patients were correctly classified. Importantly, the model maintained a low error rate, with only 17 false positives and 38 false negatives. This high specificity effectively minimizes unnecessary referrals to tertiary care while maintaining reliable detection capability. Consequently, the SVM was selected as the final model for clinical risk stratification due to its superior balance of discrimination, calibration, and net clinical benefit.

### Model interpretability

**Figure 6:**
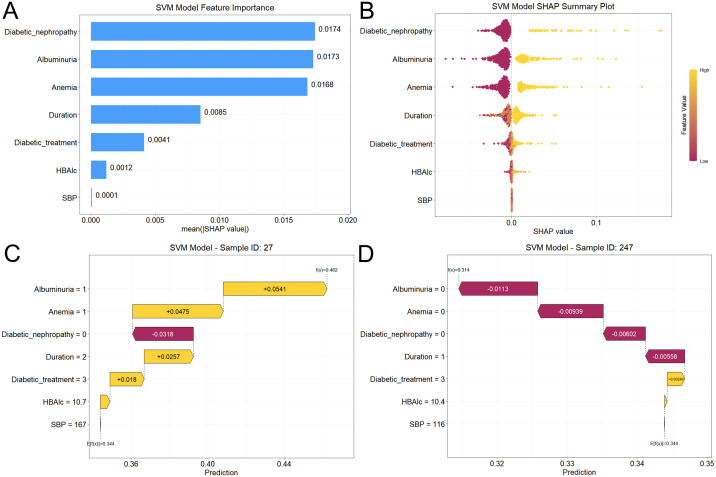
SHAP-based interpretability analysis of the SVM model. **(A)** Global feature importance ranking based on mean absolute SHAP values. **(B)** SHAP summary (beeswarm) plot. Each point represents a feature’s SHAP value magnitude and directionality, with yellow indicating higher feature values (positive impacts on VTDR risk) and red indicating lower values (negative impacts on VTDR risk). **(C, D)** SHAP waterfall plots for representative VTDR-positive and non-VTDR cases. Bar colors reflect individualized risk contributors (yellow: risk-enhancing; red: protective). SHAP, Shapley Additive Explanation; VTDR, Vision-Threatening Diabetic Retinopathy; HbA1c, Glycated Hemoglobin; SBP, Systolic Blood Pressure.

To provide clinical transparency and elucidate the internal logic of the SVM model, we employed SHAP values to quantify the feature contributions. SHAP-based feature importance ranking (**Figure 6A**) identified diabetic nephropathy, albuminuria, anemia, T2DM duration, and diabetic therapy as the five most influential factors associated with the presence of VTDR. The SHAP summary (beeswarm) plot (**Figure 6B**) provides a granular visualization of the directional impact of these features on the model’s output. Specifically, elevated levels of HbA1c and SBP, the presence of albuminuria, anemia, and diabetic nephropathy, as well as insulin treatment and prolonged T2DM duration, were positively associated with increased VTDR risk (indicated by positive SHAP values). In contrast, lower HbA1c levels, shorter T2DM duration, non-insulin treatment, and the absence of albuminuria or anemia contributed to a reduced likelihood of VTDR (indicated by negative SHAP values).

To demonstrate local interpretability, SHAP waterfall plots were used to dissect individualized risk stratification for representative cases (**Figure 6C, D**). For a representative VTDR-positive patient (**Figure 6C**), the model predicted a high probability of disease (0.462 vs. baseline 0.344). This elevation was driven primarily by risk-enhancing factors such as the presence of albuminuria (+0.054), anemia (+0.048), long-standing T2DM duration (>10 years, +0.026), and insulin-based therapy (SHAP = +0.018). Conversely, for a VTDR-negative case (**Figure 6D**), the absence of anemia (-0.011), albuminuria (-0.009), diabetic nephropathy (-0.006), and a moderate T2DM duration (5–10 years, SHAP = -0.006) acted as protective factors that substantially lowered the predicted risk.

### Clinical implementation and web-based interface

**Figure 7:**
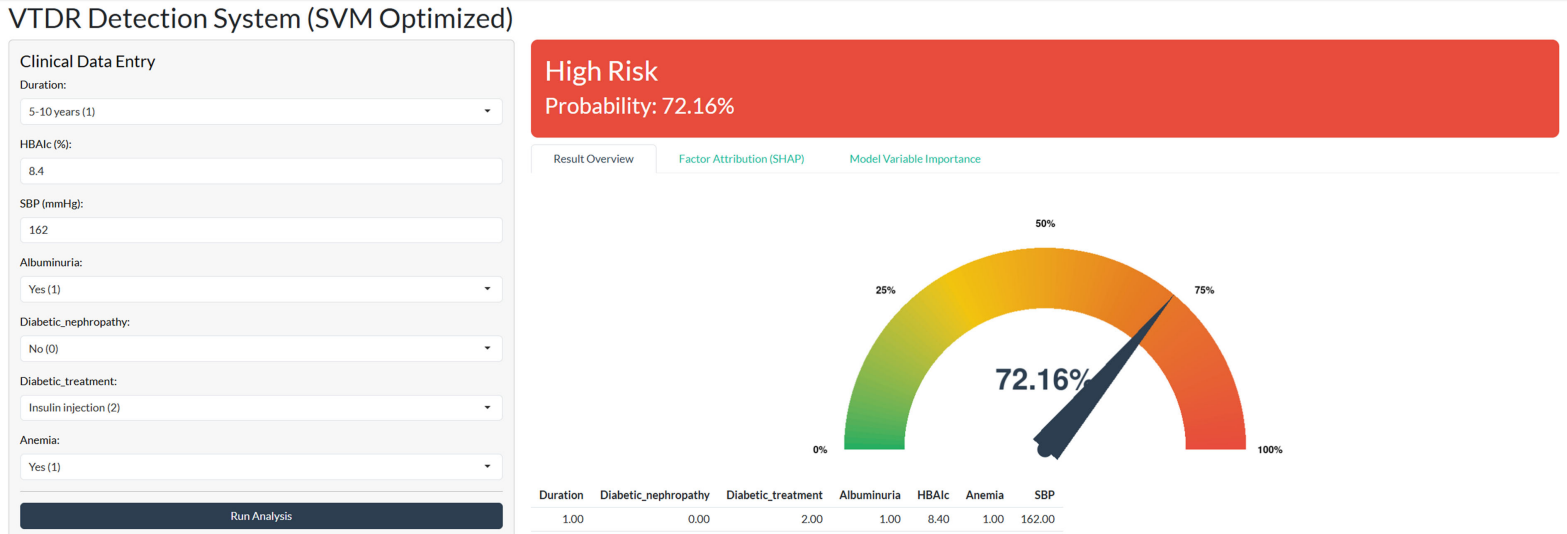
Interface and functional demonstration of the web-based SVM predictive tool. The application allows for real-time risk estimation by inputting the seven consensus clinical factors. In the example shown, the tool computes an individualized probability of VTDR presence, facilitating data-driven clinical decision support in primary care settings. HbA1c, Glycated Hemoglobin; SBP, Systolic Blood Pressure.

To facilitate practical utility in routine ophthalmological care and primary screening, the optimal detection model was integrated into a user-friendly web application. By inputting the seven identified clinical parameters, clinicians can automatically calculate an individualized probability of VTDR presence (**Figure 7**). This decision support tool is designed as a proof-of-concept to aid in risk-stratified screening and is publicly accessible at: https://pmvtdr234.shinyapps.io/60204/.

## Discussion

In this study, we developed eight ML-based detection models to identify the presence of VTDR using EMR data. Among these, the SVM algorithm outperformed the others, offering both high discriminative accuracy and clinical interpretability. This framework addresses the pressing need for scalable VTDR screening tools by synthesizing heterogeneous clinical data routinely collected during hospital visits. Unlike DL models based on fundus photography, which require high-end GPUs and large storage capacity, our tabular data approach is computationally efficient and lightweight. This makes it a cost-effective solution for integration into basic web-based platforms or EMR systems in resource-constrained primary care settings. The derived risk score, simplified for clinical applicability, facilitates the early identification of high-risk patients and enables the prioritization of preventive interventions. While this study serves as a proof-of-concept requiring external validation, it offers an evidence-based framework for implementing risk-stratified screening intervals, thereby enhancing program efficiency without compromising patient safety.

Although annual screening is widely recommended in clinical guidelines, real-world evidence from large-scale national screening programs indicates that fixed annual screening for all individuals with diabetes is often neither deliverable nor sustainable due to increasing patient volumes, limited healthcare resources, and consistently suboptimal attendance rates (30, 31). In China, the critical shortage of ophthalmologists further amplifies this challenge, limiting timely intervention for the escalating prevalence of VTDR (32). Recent advancements in modeling have demonstrated the potential to stratify VTDR risk among patients with T2DM. Ke et al. developed a nomogram model integrating clinical features to assess VTDR progression in mild DR cases, achieving an AUC of 0.730 (33). Nugawela et al. proposed risk models tailored for resource-limited settings, emphasizing feasibility in low-infrastructure environments (34). Gong et al. further validated a nomogram for VTDR assessment in T2DM cohorts, reporting an AUC of 0.690 (35). Recently, AI has emerged as a promising tool for VTDR risk stratification, offering the potential to enhance screening efficacy and prioritize high-risk patients for timely intervention (36). For instance, Bellemo et al. developed an ensemble AI model for classifying retinal color fundus images, demonstrating robust performance in detecting referable VTDR (37). Arcadu et al. further developed advanced DL algorithms to predict DR progression using fundus photographs from patients with DR (38). However, a major limitation of these image-based DL models is their reliance on high-resolution imaging infrastructure and extensive computational resources. This creates a barrier to deployment in primary care clinics where hardware is limited. In contrast, our study demonstrates that utilizing readily available tabular clinical data can achieve comparable diagnostic utility with significantly lower computational costs, offering a more accessible alternative for widespread screening.

In this study, eight ML algorithms were systematically evaluated to detect VTDR in patients with DR. The SVM model outperformed competing algorithms, demonstrating superior discrimination (AUC: 0.879), robust calibration, and clinical utility, while retaining simplicity and interpretability, which are critical advantages for clinical deployment. The strength of SVM derives from its kernel-based approach, which effectively captures non-linear interactions among risk factors without overfitting to noise, a common challenge in heterogeneous clinical datasets (39). This aligns with Jo et al., who reported that SVM achieved the highest predictive accuracy for VTDR in a South Korean T2DM cohort using longitudinal 10-year clinical data (40). Similarly, An et al. developed an SVM-based model to assess VTDR in T2DM patients with mild DR (41), further validating its robustness. These studies collectively underscore the adaptability of SVMs across diverse cohorts, even with limited sample sizes or incomplete features. Notably, Jiang and Li reported LR as the optimal model for DR prediction in their T2DM cohort (42), which contrasts with our results where LR was inferior to SVM. This discrepancy underscores the need for region-specific investigations to identify optimal ML models for VTDR detection, as algorithmic performance may vary with demographic, clinical, or data heterogeneity.

In our pursuit of an optimal screening tool, we rigorously evaluated both single-estimator algorithms (e.g., SVM, LR) and standard ensemble architectures (including Bagging [RF] and Boosting [XGB, LGB, CAT]). While ensemble methods are renowned for reducing variance, our independent testing revealed that the SVM model consistently outperformed these tree-based ensembles in terms of generalization capability and calibration. Consequently, we prioritized model parsimony over further complexity. We deliberately opted against deploying “meta-ensemble” techniques (such as Stacking or Weighted Voting classifiers). Although Stacking can marginally improve stability, it often creates an opaque “black box” structure that complicates interpretability (43). Given that our single SVM model achieved satisfactory diagnostic accuracy (AUC > 0.87), avoiding the complexity of Stacking allowed us to maintain a clear, direct linkage between clinical features and risk scores via SHAP analysis, which is paramount for clinical adoption.

To enhance the interpretability of our detection model, we integrated SHAP beeswarm and waterfall plots that provided both population- and individual-level rankings of risk factors. These tools enable clinicians to prioritize interventions for high-risk patients. During routine screening, physicians can utilize patients’ baseline clinical data alongside visualized SHAP waterfall plots to identify individualized risk profiles. This approach not only facilitates the early detection of critical VTDR risk factors but also supports the implementation of tailored preventive strategies. For instance, our analysis identified diabetes duration as an independent factor associated with VTDR. Furthermore, poor glycemic control, as reflected by elevated HbA1c levels, accelerates DR progression. Chronic hyperglycemia induces cumulative microvascular damage in the retina, promoting ischemia, inflammation, and pathological neovascularization (35, 44). Increasing evidence suggests that maintaining HbA1c below 7% significantly reduces DR progression risk (45, 46). Regarding insulin usage, our SHAP analysis identified that insulin therapy was associated with a higher probability of VTDR. It is critical to interpret this finding through the lens of “confounding by indication” rather than causality. In clinical practice, insulin is typically prescribed to patients with advanced beta-cell failure, longer disease duration, or suboptimal glycemic control despite oral agents—all of which are established risk factors for DR (47, 48). Therefore, in our detection model, insulin usage functions as a robust surrogate marker for disease severity and chronicity. The elevated SHAP value indicates that patients requiring insulin are intrinsically at higher risk due to their underlying metabolic status, not that the exogenous insulin itself induces retinopathy. Accordingly, this association highlights the need for vigilant retinal screening in insulin-treated patients, rather than suggesting any modification to their glycemic management regimen.

This study demonstrated that patients with anemia exhibited a significantly higher prevalence of VTDR. The association with DR is likely driven by insufficient retinal tissue oxygenation, a multifactorial process influenced by capillary blood flow, hemoglobin concentration, and hemoglobin oxygen affinity (49). While correcting anemia is a standard practice to improve overall quality of life, its specific role in halting DR progression remains a subject of ongoing research (50). Currently, optimizing hematological parameters is a standard of care for diabetic kidney disease, suggesting that the infrastructure and clinical pathways for anemia management in diabetic patients are well-established (51, 52). Therefore, anemia correction should be viewed as a cost-effective adjunctive strategy to reduce the hypoxic burden on the retina, potentially enhancing the efficacy of direct ocular therapies (53). Future longitudinal studies are needed to determine if raising hemoglobin levels directly mitigates retinal ischemia or if anemia is merely a systemic marker of microvascular burden. Furthermore, our analysis identified DN as an independent risk factor for VTDR. DN and VTDR share overlapping pathological mechanisms and frequently co-occur owing to analogous microvascular damage induced by chronic hyperglycemia (54). Albuminuria, a well-established biomarker of glomerular filtration barrier dysfunction (55), has been consistently linked to the presence of VTDR in recent investigations (33, 56), which is consistent with our observations. Chronic hyperglycemia precipitates systemic microvascular injury, simultaneously affecting the retinal and renal tissues, thereby establishing a clinical interplay between these complications. Consequently, integrating albuminuria testing into routine diabetes management protocols may facilitate early detection of microvascular dysfunction, enabling timely interventions to mitigate the progression of severe retinopathy in T2DM patients.

Regarding clinical utility, the deployment of our optimized model via a web-based interface demonstrates high translational potential. Performance profiling confirmed that the system achieves negligible inference latency (< 1 second per request), primarily due to the computational efficiency of processing low-dimensional tabular data compared to high-resolution imaging models. This sub-second response time is critical, ensuring seamless integration into time-sensitive primary care workflows without disrupting the patient consultation process. To further safeguard against data entry errors—a common challenge in fast-paced clinical settings—the web interface incorporates strict input validation logic based on biological plausibility. We implemented predefined range checks for continuous variables (e.g., restricting HbA1c to 3.0%–20.0% and SBP to 50–250 mmHg) and format constraints for categorical inputs. These “digital guardrails” effectively filter out typos or physiologically impossible values that could otherwise compromise diagnostic accuracy. Furthermore, aligning with the growing demand for transparent AI in medicine, the tool moves beyond a binary output by including dedicated modules for “Factor Attribution” and “Variable Importance”. By visualizing the contribution of individual patient factors (e.g., highlighting that a specific patient’s high risk is driven by insulin usage and anemia), the tool supports clinical decision-making beyond a traditional “black box” output, thereby fostering clinician trust and facilitating personalized patient counseling.

## Limitations

This study has several limitations that must be acknowledged. First, the study was conducted at a single medical center involving exclusively Chinese patients. The lack of external validation limits the generalizability of our findings to other racial/ethnic groups and clinical settings. Consequently, the current model should be viewed as a proof-of-concept for screening rather than a fully actionable clinical tool. Large-scale, multicenter, international validation studies are warranted to assess the reproducibility and broader applicability of this framework before clinical deployment. Second, we acknowledge the potential influence of selection bias inherent to hospital-based studies. The prevalence of VTDR in our cohort (nearly 37%) exceeds typical reported rates (≤ 25%) (33, 49, 57), reflecting the referral patterns of a tertiary ophthalmic center. While this enriched dataset facilitated the robust learning of severe disease features, users should note that the model’s probability calibration—specifically precision (positive predictive value)—may require adjustment (recalibration) when applied to low-prevalence primary care populations. Third, the retrospective, cross-sectional design introduced inherent limitations regarding temporal ambiguity. Since the data represents a single time point, causal relationships cannot be established. For instance, associations between treatment modalities (e.g., insulin) and VTDR should be interpreted as confounding by indication (markers of disease severity) rather than causative mechanisms. Furthermore, the potential for residual confounding from unmeasured factors (e.g., genetic susceptibility, socioeconomic status) remains. Fourth, regarding data quality, we excluded variables with more than 20% missing data to ensure modeling integrity (**Supplementary Table 1**). We acknowledge that this rigorous filtering might have resulted in the loss of specific biomarkers that could possess prognostic value. Prospective studies with standardized, synchronized data collection are required to validate these findings. Fifth, regarding the modeling strategy, while we evaluated standard ensemble methods (e.g., RF, XGB), we deliberately prioritized model parsimony and did not pursue complex “meta-ensemble” architectures (such as Stacking). While this decision ensured the transparency of our SHAP-based interpretability, future work could investigate if stacking multiple weak learners yields marginal gains in screening accuracy, despite the potential cost of model simplicity and explainability. Finally, the absence of long-term follow-up data precluded the assessment of longitudinal progression. Future studies with defined time-to-event analyses are required to transition this framework from a current detection tool to a true prognostic model capable of predicting future risk.

## Conclusion

In conclusion, this study developed an explainable ML model to detect the presence of VTDR among patients with DR using readily accessible clinicopathological parameters extracted from routine EMRs. Among the eight evaluated algorithms, the SVM model demonstrated superior diagnostic accuracy for VTDR. Critically, by leveraging low-dimensional tabular data rather than computationally intensive retinal imagery, this framework overcomes the infrastructure barriers of DL models, offering a scalable and accessible solution for resource-constrained primary care settings. Furthermore, the integration of SHAP-based visualization tools elucidates individual risk profiles, offering a novel framework to enhance clinical decision-making. While large-scale external validation is required, this study serves as a strong proof-of-concept that bridges the gap between computational models and practical healthcare applications, supporting early intervention strategies to improve patient outcomes.

## Data Availability

The raw data supporting the conclusions of this article will be made available by the authors, without undue reservation.
