# Supplementary material for: An interpretable machine learning model for detecting vision-threatening diabetic retinopathy among patients with diabetic retinopathy: a web-based cross-sectional study
Source: Front Endocrinol (Lausanne). 2026 Mar 4;17:1776188. doi: 10.3389/fendo.2026.1776188 (PMC12997098; doi:10.3389/fendo.2026.1776188)
Supplement: Supplementary file 1 [file DataSheet1.docx]

**Supplementary Table S1.** Variables excluded from the analysis due to high missing rate (>20%).

| Category | Variables | Missing rate (%) | Rationale for exclusion |
| --- | --- | --- | --- |
| Demographics | Waist circumference | 74.5% | Routine measurement is not performed in all clinic visits. |
|  | Waist-to-Hip ratio | 74.5% | Derived from missing waist circumference data. |
|  | Occupation | 52.6% | Incomplete documentation in the retrospective Electronic Medical Records (EMR). |
| Metabolic & Glycemic markers | Fasting insulin | 65.4% | Not routinely screened for all T2DM outpatients during standard follow-up. |
|  | Serum C-peptide | 47.7% | Primarily used for initial beta-cell function assessment or T1DM differentiation; not routinely quantified in established T2DM follow-up visits. |
|  | 2-hour postprandial glucose (2h-PG) | 36.9% | Timing of sample collection is difficult to standardize in retrospective EMR data; HbA1c is preferred for long-term glycemic monitoring. |
| Renal function markers | 24-hour urinary protein | 81.2% | Cumbersome collection process often leads to poor patient compliance. |
| Vascular & inflammatory markers | Homocysteine (Hcy) | 29.2% | Specific risk marker for cardiovascular disease; not included in the standard biochemical panel for all diabetic patients. |
|  | Fibrinogen | 21.7% | Coagulation marker typically assessed only in patients with specific cardiovascular or thrombotic indications, rather than routine diabetes screening. |

**Supplementary Table S2.** Explanation of the machine learning algorithms.

| Algorithms | Explanations |
| --- | --- |
| Logistic Regression (LR) | Models the relationship between a binary outcome and predictor variables via log-odds, which are converted to probabilities. Offers simplicity and interpretability through coefficients. Assumes linearity between predictors and log-odds. |
| Decision Tree (DT) | Hierarchical splitting of data into nodes based on feature thresholds to classify outcomes. Intuitive visualization but prone to overfitting. Key Feature: Captures nonlinear relationships without strict assumptions. |
| Random Forest (RF) | Ensemble of decorrelated decision trees trained via bootstrap aggregation (bagging). Reduces overfitting and variance by averaging predictions. Strength: Robust to noise; provides feature importance metrics. |
| Decision Tree (DT) | Identifies optimal hyperplanes in high-dimensional space to separate classes. Uses kernel tricks (e.g., polynomial, radial) for nonlinear data. Advantage: Effective in small-sample, high-dimensional settings. |
| Neural Network (NN) | Inspired by biological neurons, with interconnected layers (input, hidden, output) processing data through activation functions. Use Case: Complex pattern recognition (e.g., imaging) but requires large datasets. |
| Extreme Gradient Boosting (XGB) | Gradient-boosted trees built sequentially, correcting errors from prior models with regularization. Key Benefit: Handles missing data and prevents overfitting; outperforms traditional boosting (e.g., AdaBoost) in speed/accuracy. |
| Light Gradient Boosting Machine (LGBM) | Optimized gradient-boosting framework using histogram-based binning and leaf-wise tree growth. Advantage: Faster training and lower memory usage than XGBoost, ideal for large-scale datasets. |
| CatBoosting (CAT) | Gradient-boosting variant with native handling of categorical features via ordered boosting. Strength: Minimal preprocessing; robust to noisy categorical data. |

**Supplementary Table S3.** The best-tuned hyperparameters for model development.

| Model | Key Parameters |
| --- | --- |
| Logistic Regression (LR) | - Alpha: [0.01, 0.02, 1]  - Lambda: [0.01, 0.05, 0.7] |
| Decision Tree (DT) | - Complexity parameter (cp): [0.01, 0.03, 0.05, 0.08]  - Maximum depth (maxdepth): [5, 8, 10]  - Minimum split (minsplit): [15, 20, 30]  - Minimum bucket (minbucket): [8, 10, 15] |
| Random Forest (RF) | - Number of features to try per tree (mtry): [2, 4]  - Number of trees (ntree): [400, 500]  - Minimum size of terminal nodes (nodesize): [40, 45]  - Maximum nodes (maxnodes): [30, 40]  - Sample size (sampsize): 300 |
| Support Vector Machine (SVM) | - Cost (C): [4, 8, 16, 32]  - RBF kernel parameter (sigma): [0.005, 0.008, 0.01] |
| Neural Network (NNet) | - Number of hidden layer nodes (size): [1, 3, 5]  - Weight decay (decay): [0.1, 0.5, 1, 3] |
| XGBoost (XGB) | - Maximum depth (max_depth): [2, 4]  - Minimum child weight: [5, 8]  - Learning rate (eta): 0.01  - Subsample ratio: 0.5  - Column sampling rate (colsample_bytree): 0.5  - Minimum loss reduction (gamma): 1  - L2 regularization (reg_lambda): 10 |
| Light Gradient Boosting Machine (LGBM) | - Number of leaves (num_leaves): [2, 4]  - L2 regularization (reg_lambda): [1.0, 2.0]  - Subsample ratio: [0.5, 0.6]  - Column sampling rate (colsample_bytree): 0.8  - Learning rate: 0.05 |
| CatBoosting (CAT) | - Tree depth (depth): 2  - Number of iterations: 800  - Learning rate: 0.01  - Feature fraction (rsm): 0.4  - Random strength: 30  - L2 regularization (reg_lambda): 50  - Subsample: 0.5  - Scale weight positive (weight_pos): 1.5 |

**Supplementary Table S4.** Baseline characteristics of patients with retinopathy (N = 1,124).

| Parameters | Total  (N=1,124) | Training cohort (N=787) | Testing cohort  (N=337) | Statistics | *p*-value |
| --- | --- | --- | --- | --- | --- |
| Age (years) | 67.0 [57.0, 75.0] | 67.0 [57.0, 75.0] | 68.0 [56.0, 76.0] | -0.715 | 0.474 |
| Gender |  |  |  |  |  |
| Male | 686 (61.0%) | 479 (60.9%) | 207 (61.4%) | 0.031 | 0.860 |
| Female | 438 (39.0%) | 308 (39.1%) | 130 (38.6%) |  |  |
| BMI (kg/m^2^) | 23.9 [20.9, 28.4] | 23.9 [20.8, 28.4] | 23.9[21.1, 28.3 | -0.249 | 0.803 |
| Residence |  |  |  |  |  |
| Rural | 481 (42.8%) | 340 (43.2%) | 141 (41.8%) | 0.179 | 0.672 |
| Urban | 643 (57.2%) | 447 (56.8%) | 196 (58.2%) |  |  |
| Education level |  |  |  |  |  |
| Middle school or below | 640 (56.9%) | 459 (58.3%) | 181 (53.7%) | 2.048 | 0.152 |
| High school or above | 484 (43.1%) | 328 (41.7%) | 156 (46.3%) |  |  |
| Duration of diabetes |  |  |  |  |  |
| <5 years | 147 (13.1%) | 106 (13.5%) | 41 (12.2%) | 0.354 | 0.838 |
| 5-10 years | 461 (41.0%) | 321 (40.8%) | 140 (41.5%) |  |  |
| >10 years | 516 (45.9%) | 360 (45.7%) | 156 (46.3%) |  |  |
| Smoking status |  |  |  |  |  |
| Never | 737 (65.6%) | 524 (66.6%) | 213 (63.2%) | 1.326 | 0.515 |
| Former | 179 (15.9%) | 120 (15.2%) | 59 (17.5%) |  |  |
| Current | 208 (18.5%) | 143 (18.2%) | 65 (19.3%) |  |  |
| Alcohol consumption |  |  |  |  |  |
| Never | 662 (58.9%) | 465 (59.1%) | 197 (58.5%) | 0.066 | 0.967 |
| Former | 225 (20.0%) | 156 (19.8%) | 69 (20.5%) |  |  |
| Current | 237 (21.1%) | 166 (21.1%) | 71 (21.1%) |  |  |
| Family history of T2DM | 547 (48.7%) | 395 (50.2%) | 152 (45.1%) | 2.444 | 0.118 |
| Eye surgery history | 283 (25.2%) | 197 (25.0%) | 86 (25.5%) | 0.030 | 0.863 |
| Hyperlipidemia | 353 (31.4%) | 239 (30.4%) | 114 (33.8%) | 1.311 | 0.252 |
| Cardiovascular disease | 277 (24.6%) | 188 (23.9%) | 89 (26.4%) | 0.808 | 0.369 |
| Anemia^a^ | 362 (32.2%) | 244 (31.0%) | 118 (35.0%) | 1.739 | 0.187 |
| Stroke | 129 (11.5%) | 92 (11.7%) | 37 (11.0%) | 0.117 | 0.732 |
| Diabetic nephropathy | 193 (17.2%) | 142 (18.0%) | 51 (15.1%) | 1.405 | 0.236 |
| Diabetic peripheral neuropathy | 148 (13.2%) | 96 (12.2%) | 52 (15.4%) | 2.156 | 0.142 |
| Lower extremity arterial disease | 93 (8.3%) | 71 (9.0%) | 22 (6.5%) | 1.933 | 0.164 |
| High myopia | 147 (13.1%) | 100 (12.7%) | 47 (13.9%) | 0.319 | 0.572 |
| Albuminuria | 408 (36.3%) | 292 (37.1%) | 116 (34.4%) | 0.734 | 0.392 |
| Diabetic treatment |  |  |  |  |  |
| Dietary control | 192 (17.1%) | 132 (16.8%) | 60 (17.8%) | 1.901 | 0.593 |
| Oral drugs | 358 (31.9%) | 243 (30.9%) | 115 (34.1%) |  |  |
| Insulin injection | 301 (26.8%) | 218 (27.7%) | 83 (24.6%) |  |  |
| Oral drugs + insulin | 273 (24.3%) | 194 (24.7%) | 79 (23.4%) |  |  |
| SBP (mmHg) | 136.0 [118.0, 154.0] | 138.0 [117.0, 156.0] | 135.0 [118.0, 153.0] | -0.236 | 0.814 |
| DBP (mmHg) | 84.0 [75.0, 93.8] | 83.0 [73.0, 93.0] | 84.0 [75.0, 94.0] | -1.591 | 0.112 |
| Laboratory parameters |  |  |  |  |  |
| FPG (mmol/L) | 7.4 [6.4, 8.5] | 7.4 [6.4, 8.9] | 7.3 [6.4, 8.4] | -0.273 | 0.785 |
| HbA1c (%) | 8.0 [6.2, 9.8] | 7.8 [6.1, 9.5] | 8.1 [6.4, 9.8] | -1.385 | 0.166 |
| BUN (mmol/L) | 5.7 [4.7, 6.6] | 5.7 [4.8, 6.6] | 5.6 [4.8, 6.6] | -0.518 | 0.604 |
| SCr (μmol/L) | 78.0 [61.0, 94.0] | 78.0 [62.0, 93.5] | 78.0 [62.0, 93.5] | -0.090 | 0.928 |
| UA (μmol/L) | 325.0 [265.3, 390.8] | 326.0 [268.5, 385.5] | 325.0 [263.0, 391.0] | -0.301 | 0.763 |
| TC (mmol/L) | 5.2 [4.4, 5.9] | 5.3 [4.4, 6.0] | 5.2 [4.4, 5.8] | -1.080 | 0.280 |
| TG (mmol/L) | 1.6 [0.7, 2.5] | 1.7 [0.8, 2.5] | 1.6 [0.7, 2.5] | -0.832 | 0.405 |
| HDL-C (mmol/L) | 1.3 [1.1, 1.5] | 1.3 [1.1, 1.5] | 1.3 [1.0, 1.5] | -0.052 | 0.959 |
| LDL-C (mmol/L) | 3.0 [2.8, 3.2] | 3.0 [2.8, 3.2] | 3.0 [2.8, 3.2] | -0.443 | 0.658 |
| Alb (g/L) | 38.0 [33.0, 44.0] | 38.0 [33.0, 44.0] | 38.0 [33.0, 44.0] | -0.526 | 0.599 |
| CRP (mg/L) | 4.2 [2.3, 5.6] | 4.0 [2.3, 5.5] | 4.2 [2.3, 5.7] | -0.104 | 0.917 |
| AST (U/L) | 28.0 [22.0, 35.0] | 29.0 [22.0, 35.0] | 28.0 [22.0, 35.0] | -0.555 | 0.579 |
| ALT (U/L) | 32.0 [26.0, 37.0] | 32.0 [26.0, 37.0] | 32.0 [27.0, 37.0] | -0.847 | 0.397 |
| WBC (10^9^/L) | 7.0 [5.5, 8.6] | 6.9 [5.6, 8.6] | 7.0 [5.5, 8.7] | -0.153 | 0.878 |
| PLT (10^9^/L) | 220.0 [159.0, 284.0] | 224.0 [159.0, 291.0] | 215.0 [159.0, 282.0] | -1.275 | 0.202 |
| LYM (10^9^/L) | 2.4 [1.6, 3.2] | 2.3 [1.5, 3.1] | 2.4 [1.6, 3.3] | -1.211 | 0.226 |
| MONO (10^9^/L) | 0.6 [0.4, 0.8] | 0.6 [0.4, 0.9] | 0.6 [0.4, 0.8] | -0.372 | 0.710 |
| NEUT (10^9^/L) | 4.3 [3.1, 5.5] | 4.3 [3.2, 5.5] | 4.3 [3.1, 5.5] | -0.206 | 0.837 |
| NLR | 1.9 [1.2, 2.9] | 2.0 [1.3, 3.0] | 1.8 [1.2, 2.8] | -1.395 | 0.163 |
| VTDR | 415 (36.9%) | 292 (37.1%) | 123 (36.5%) | 0.037 | 0.847 |

Note. ^a^Anemia was defined according to WHO criteria: Hemoglobin <130 g/L for men and <120 g/L for women. To ensure temporal accuracy, this classification was determined dynamically using the specific hemoglobin measurement obtained within the 15-day window immediately preceding the fundus photography. This approach ensures a time-adjusted assessment of the patient’s hematological status concurrent with the retinal examination.

Values are presented as median [Interquartile range] for continuous variables and N (%) for categorical variables.

**Abbreviations:** BMI: Body Mass Index; T2DM: Type 2 Diabetes Mellitus; SBP: Systolic Blood Pressure; DBP: Diastolic Blood Pressure; FPG: Fasting Plasma Glucose; HbA1c: Glycated Hemoglobin; BUN: Blood Urea Nitrogen; SCr: Serum Creatinine; UA: Uric Acid; TC: Total Cholesterol; TG: Triglycerides; HDL-C: High-Density Lipoprotein Cholesterol; LDL-C: Low-Density Lipoprotein Cholesterol; Alb: Albumin; CRP: C-Reactive Protein; AST: Aspartate Aminotransferase; ALT: Alanine Aminotransferase; WBC: White Blood Cell Count; PLT: Platelet Count; LYM: Lymphocyte Count; MONO: Monocyte Count; NEUT: Neutrophil Count; NLR: Neutrophil-to-Lymphocyte Ratio

**Supplementary Figure S1.**


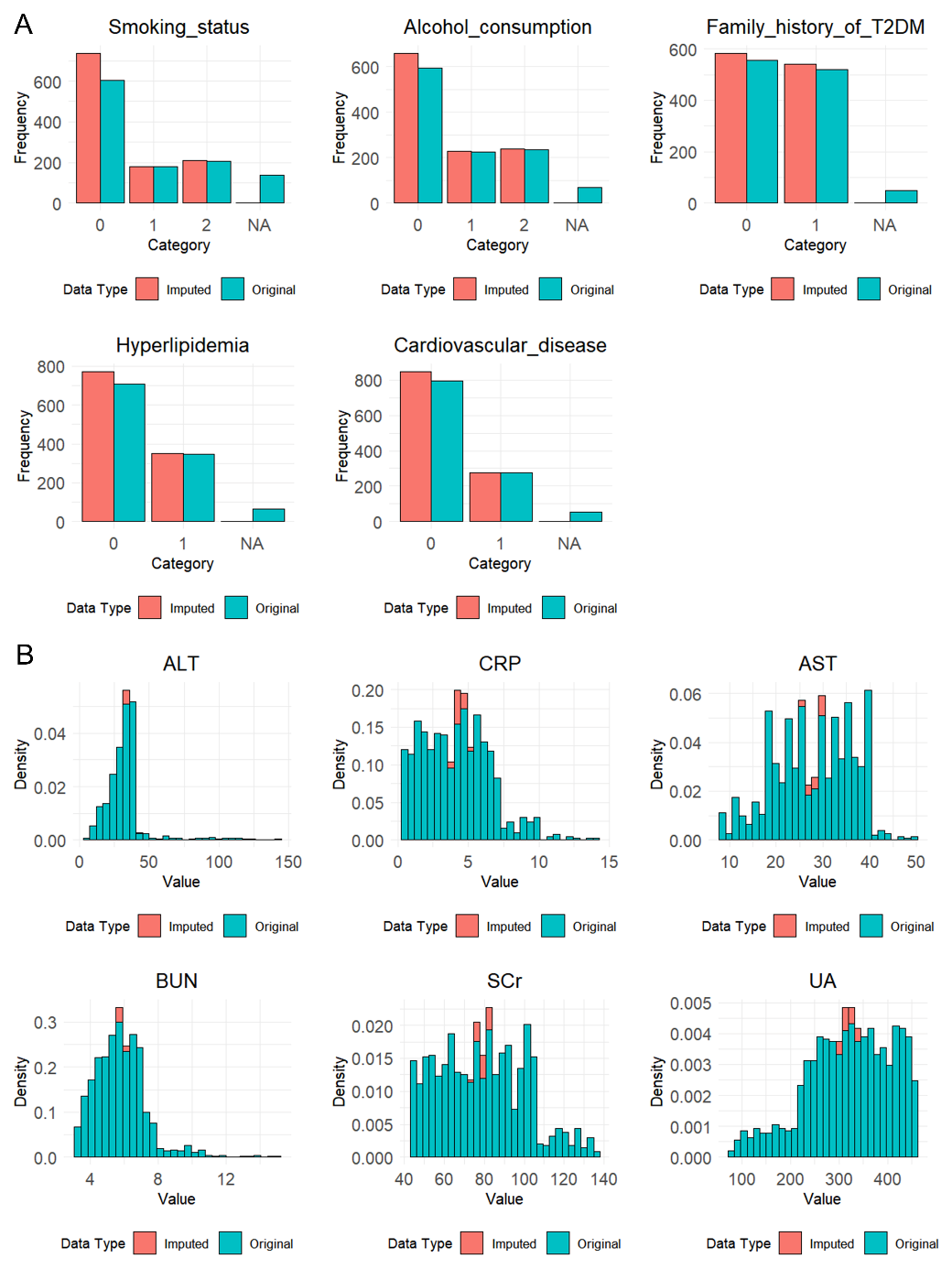


**Supplementary Figure S1.** Comparison of missing data before and after imputation among category (A) and continuous variables (B).
